# Identifying novel sphingosine kinase 1 inhibitors as therapeutics against breast cancer

**DOI:** 10.1080/14756366.2019.1692828

**Published:** 2019-11-22

**Authors:** Faez Iqbal Khan, Dakun Lai, Razique Anwer, Iffat Azim, Mohd Kalim Ahmad Khan

**Affiliations:** a School of Electronic Science and Engineering, University of Electronic Science and Technology of China, Chengdu, China; b Department of Pathology, College of Medicine, Imam Mohammad ibn Saud Islamic University, Riyadh, Saudi Arabia; c Department of Bioengineering, Faculty of Engineering, Integral University, Lucknow, India

**Keywords:** SphK1, breast cancer, molecular docking, MD simulation, MMPBSA calculations

## Abstract

Sphingosine kinase 1 (SphK1) is a promising therapeutic target against several diseases including mammary cancer. The aim of present work is to identify a potent lead compound against breast cancer using ligand-based virtual screening, molecular docking, MD simulations, and the MMPBSA calculations. The LBVS in molecular and virtual libraries yielded 20,800 hits, which were reduced to 621 by several parameters of drug-likeness, lead-likeness, and PAINS. Furthermore, 55 compounds were selected by ADMET descriptors carried forward for molecular interaction studies with SphK1. The binding energy (Δ*G*) of three screened compounds namely ZINC06823429 (–11.36 kcal/mol), ZINC95421501 (–11.29 kcal/mol), and ZINC95421070 (–11.26 kcal/mol) exhibited stronger than standard drug PF-543 (–9.9 kcal/mol). Finally, it was observed that the ZINC06823429 binds tightly to catalytic site of SphK1 and remain stable during MD simulations. This study provides a significant understanding of SphK1 inhibitors that can be used in the development of potential therapeutics against breast cancer.

## 1. Introduction

Breast cancer is the most commonly diagnosed cancer among women worldwide. A majority of patients diagnosed with breast cancer go on to develop symptoms of post-traumatic stress disorder, and in most of these cases the symptoms persist for at least a year^1^. Pain, fatigue, arm morbidity, and postmenopausal symptoms were among the most common symptoms are reported by breast cancer patients^2^. The recognition and treatment of these symptoms are main issues since these symptoms impair health-related quality of life. It can strike at any age, but it is commonly diagnosed in middle-aged women^3^. According to International Agency for Research on Cancer (IARC) of World Health Organization (WHO), there were 2.09 million cases of breast cancer reported globally, out of which 627,000 deaths have been occurred in 2018. The estimated numbers of new cases of breast cancer in 2018 in the USA were 268,670 with total 41,400 deaths^4^. The breast cancer mortality rates have been declined in the past few years owing to early diagnosis and advancement in treatments^5^.

The present medication such as endocrine therapy for the different subtypes of breast cancer is utilised for the treatment and prevention of oestrogen-receptor-positive (ER+) breast carcinoma^6^. The ER + and human epidermal growth factor receptor 2 (HER-2) sub types breast cancer are treated by tamoxifen and monoclonal antibody, respectively^7–10^. Endocrine therapy is the most effective and least toxic treatment for breast cancer, but it poses challenges. The major challenges are late recurrence, drug resistance, painful injection, and lack of compliance^11^. The increase in the utilisation of endocrine agents has brought about the improvement of procedures to prevent and eradicate mammary cancer. About 30% of both ER + and progesterone-receptor-positive (PR+) tumours are not susceptible to endocrine treatment^12^. There are limitations in predicting the efficacy of endocrine treatment for hormone receptor expression alone. Therefore, the combination therapies are utmost needed to reduce the chemo-resistance.

It has been reported that sphingosine-1-phosphate (S1P), a sphingolipid bioactive molecule that mediates various biological processes plays a key role in breast carcinoma^13^. The process starts when S1P is produced intracellularly by two sphingosine kinases such as sphingosine kinase 1 (SphK1) and SphK2 with autocrine and paracrine action called “inside out signaling”^14^^,^^15^. The action of SphK1 is mediated by few developmental factors such as oestradiol (E2), epidermal growth factor (EGF), vitamins and cytokines IL1, and IL6^14^^,^^16^. The active transport of S1P from the cell takes place with the help of oestradiol (E2) via ATP binding cassette transporter A1 (ABC A1), ATP binding cassette transporter C1 (ABC C1), and ATP binding cassette transporter G2 (ABC G2)^17^. The S1P regulates various biological processes such as cell development, anti-apoptosis, intrusion, angiogenesis, and expansion after attaching to five selective G protein-coupled receptor (GPCRs) located on the plasma membrane (S1P1-5). This in turn stimulates ERK1/2 prompting downstream signalling that is a crucial step for metastasis of breast cancer^14^. Apart from regulating ERK1/2 pathway, it also mediates PI3K/AKT signalling pathway^18^.

It has been well documented that high expression of SphK1 is associated with different subsets of breast carcinoma such as ER+, HER 2, and TNBC, thereby resulting the worse prognosis and resistance to available antitumour therapies^19^^,^^20^. Various inhibitors of SphK1 such as PF-543, SK1-5c, Belanocarpol, RB-005, and VPC96091 have been designed so far, but to identify the finest one having plausible bioavailability, pharmacokinetic, and pharmacodynamic properties is yet to be developed^16^^,^^21–23^.

The main objective of present work is to identify a potent lead compound against breast cancer using several computational approaches such as the ligand-based virtual screening (LBVS)^24–26^, molecular docking^27–29^, molecular dynamics (MD) simulations^30–33^, and the molecular mechanics (MM) Poisson–Boltzmann surface area (MMPBSA) calculations^34^. The molecular interaction studies of predicted lead molecules with target protein SphK1 were also carried out in order to check the strength of drug binding. The results were further compared with known inhibitor PF-543^35^, and the best drug was identified. This study may provide valuable insight leading to improved treatment of patients with breast cancer.

## 2. Methodology

### 2.1. SphK1 protein preparations

The 3D structure of SphK1 in complex with PF-543 (4V24) was obtained from RCSB Protein Data Bank (http://www.rcsb.org)^36^. The coordinates of heteroatoms were removed to prepare the protein for molecular docking. The energy of SphK1 was minimised by applying the CHARMm force field. The active site of SphK1 was located for ligand binding where the inhibitor PF-543 was co-crystallised^36^.

### 2.2. Ligand based virtual screening

SwissSimilarity^37^, a novel web-based tool that offers ultra-high-throughput virtual screening from various repositories of chemical compounds such as DrugBank^38^, Ligand Expo^39^, ChEMBL^40^, ChEBI^41^, GLASS^42^, the Human Metabolome Database (HMDB)^43^, and ZINC databases^44^ was used to perform LBVS. The predictions were based on five different 2D and 3D search techniques, namely molecular fingerprints (FPs), electroshape, spectrophores, shape IT, and align IT. The screening by these approaches are based on similarity score such as 1 for similar and 0 for dissimilar compounds.

### 2.3. Drug likeness, lead likeness, and PAINS

Different filters were applied to predict hits based on Lipinski’s RO5^45^^,^^46^, Veber^47^, Ghose^48^, Egan^49^, Muegge^50^, lead likeness, and pan-assay interference compounds analysis (PAINS)^51^. In RO5, the attributes commonly found in orally active drug molecules of molecular weight (≤500 Da), hydrogen bond acceptor (≤10), hydrogen bond donor (5≤), and log *P* (≤5). Veber et al. improved the RO5 with the addition of two more properties including the number of rotatable bonds (≤10) and PSA (≤140 Å^2^).

Moreover, the RO5 fails to explain the basic highlights found in drugs or non-drugs. Therefore, Ghose et al. expanded this work in view of processed physicochemical properties. They set up the accompanying criteria such as *A* log *P* (–0.4 to 5.6), molar refractivity (40–130), molecular weight (160–480), and the number of atoms (20–70). Further, Muegge et al. assigned a score due to the presence of structural fragments on a molecule that are typically found in drugs. The molecules with a score ranging from 2 to 7 were classified as drugs. Two main criteria namely *S*Log*P* (≤6) and TPSA (≤132 Å^2^) were further included in Egan filtration. All the drug likeness, lead likeness, and pan assay interference studies of ligands and standard molecule were carried out using SwissADME tool^52^.

### 2.4. ADME and toxicity prediction

Absorption, distribution, metabolism, and excretion (ADME) and toxicity assessment are very important part of leads selection. The ADME and toxicity parameters of all compounds obtained after filtration on various parameters of drug likeness properties were checked using TOxicity Prediction by Komputer Assisted Technology (TOPKAT) module of Discovery studio 2.5. This tool predicts the toxicity using weight of the evidence parameter of carcinogenicity as per the Food and Drug Administration (FDA) and National Toxicology Program (NTP) animal model guidelines.

### 2.5. Molecular docking studies

AutoDock 4.2^53^ molecular docking software was used to identify the most probable binding pose and molecular interactions of inhibitors into the active pocket of SphK1. In this trend, we addressed protein preparation, chain selection, and non-protein parts deletion with Discovery Studio Visualizer 2.5. For SphK1, a grid was set up with 60 × 60 × 60 points, and docking was performed by using default parameters. The ligands were docked into the active pocket of SphK1 where the inhibitor PF-543 was present^36^. The proper orientations of docked molecules in SphK1 were selected. The compounds having least Δ*G* values were treated as strong binding affinity with the target protein.

### 2.6. Molecular dynamics simulation

MD simulations were performed on SphK1, SphK1–PF-543, SphK1–ZINC06823429, SphK1–ZINC95421070, and SphK1–ZINC95421501 at 300 K at the MM level executed by GROMACS 5.1.2^54^ utilising the GROMOS96 43a1 force-field. The ligands were extracted from the docked complexes utilising *gmx grep* module. The topology and force-field parameter files of the ligands were obtained by PRODRG server^55^. The charges in the topology file were manually corrected. The topologies were generated for SphK1 utilising *pdb2gmx* modules of gromacs and PF-543, ZINC06823429, ZINC95421070, and ZINC95421501 using PRODRG server were merged together.

The extra ligand atoms were combined in the complex topologies with default parameters. The SphK1, SphK1–PF-543, SphK1–ZINC06823429, SphK1–ZINC95421070, and SphK1–ZINC95421501 were soaked in a cubic box of water molecules with a dimension of 10 Å, i.e. setting the edge 10 Å from the molecule periphery utilising the *gmx editconf* module for creating boundary conditions and *gmx solvate* module for solvation. The Simple Point Charge (spc216) water model was used to solvate the system. The charges on the SphK1, SphK1–PF-543, SphK1–ZINC06823429, SphK1–ZINC95421070, and SphK1–ZINC95421501 complexes were neutralised by addition of Na^+^ and Cl^–^ ions using *gmx genion* module to maintain neutrality, preserving a physiological concentration of 0.15 M. Further, energy groups were used to examine the interaction of SphK1 with PF-543, ZINC06823429, ZINC95421070, and ZINC95421501. The system was then minimised using 1500 steps of steepest descent. Finally, system temperatures were raised from 0 to 300 K during their equilibration period (100 ps) at a constant volume under periodic boundary conditions.

The systems were equilibrated using NVT ensemble (constant number of particles, volume, and temperature at 100 ps) and NPT ensemble (constant number of particles, pressure, and temperature at 100 ps). In both cases, the C^α^ backbone atoms of the original crystal structure were limited with all other atoms permitted to move freely. After the equilibration phase, the particle-mesh was applied following Ewald method. The resulting path was analysed utilising *gmx energy*, *gmx rms*, *gmx confirms*, *gmx rmsf*, *gmx gyrate*, *make_ndx*, *gmx hbond*, *gmx do_dssp*, *gmx covar*, *gmx anaeig*, *gmx sham*, and *gmx sasa* utilities of GROMACS. The plots of the 3D models were readied utilising PyMol and VMD (Visual Molecular Dynamics)^56^. The details of MD simulation method were previously described^57–61^.

### 2.7. MMPBSA calculation

Molecular mechanics Poisson–Boltzmann surface area, a strategy to appraise interaction free energies was used to study bimolecular interactions. The binding energy was calculated utilising MMPBSA protocols implemented in the *g_mmpbsa* package^62^. A protein–ligand snapshot of MD trajectories between 40 and 50 ns was extracted from the stable region of every complex. The aim was to incorporate high throughput MD simulations with their binding energy calculations. The binding energies were figured utilising following equation:

(1) $$\Delta G_{\text{Binding}}=G_{\text{Complex}}-\left(G_{\text{Protein}}+G_{\text{Ligand}}\right)$$

where *G*_Complex_, *G*_Protein_, and *G*_Ligand_ signify the total free energy of the binding complex, total free energies of the individual protein, and total free energies of the individual ligand, respectively. Besides, the free energy for each individual entity can be given by:

(2) $$G_{x}=〈E_{\text{MM}}〉-TS+〈G_{\text{solvation}}〉$$

where “x” is the protein or ligand or protein–ligand complex. *E*_MM_ is the average MM potential energy in a vacuum. Temperature (*T*) and entropy (*S*) jointly denote the entropic contribution to the free energy in a vacuum and the *G*_solvation_ is the free energy of solvation. The vacuum potential energy (*E*_MM_) is the sum of bonded as well as non-bonded interactions, and it is calculated based on the MM force-field parameters.

(3) $$E_{\text{MM}}=E_{\text{bonded}}+E_{\text{non}-\text{bonded}}=E_{\text{bonded}}+\left(E_{\text{vdW}}+E_{\text{elec}}\right)$$

where *E*_bonded_ is bonded interactions consisting of bond, angle, dihedral, and improper interactions. The non-bonded interactions (*E*_non-bonded_) include both electrostatic (*E*_elec_) and van der Waals (*E*_vdW_) interactions and are modelled using a Coulomb and Lennard-Jones (LJ) potential function, respectively. The free energy of solvation is well defined by the energy required to transfer a solute from vacuum into the solvent. In the MMPBSA method, it is calculated using an implicit solvent model. The solvation free energy (*G*_solvation_) is defined as:

(4) $$G_{\text{solvation}}=G_{\text{polar}}+G_{\text{non}-\text{polar}}$$

where *G*_polar_ and *G*_non-polar_ are the electrostatic and non-electrostatic contributions to the solvation free energy, respectively.

## 3. Results

### 3.1. Ligand-based virtual screening

**Figure 1.**
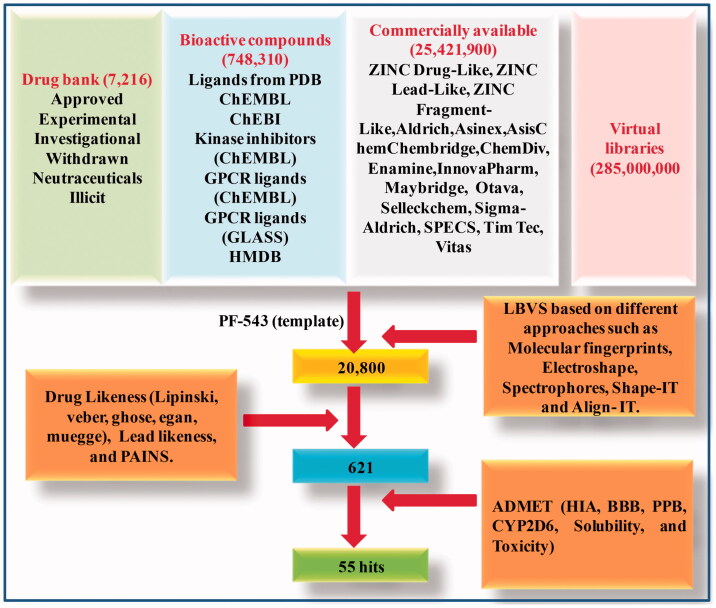
The flowchart showing the ligand-based virtual screening of compounds.

LBVS approach was employed to filter compounds so as to build the SphK1 inhibitor library. The PF-543, a potent inhibitor of SphK1 was used as input molecule to extract small molecules similar to SphK1 inhibitor from the constructed libraries using virtual screening strategies. LBVS identified 20,800 hits from various molecular and virtual libraries including DrugBank, Ligand Expo, ChEMBL, ChEBI, GLASS, HMDB, and ZINC databases as shown in Figure 1.

### 3.2. Drug likeness, lead likeness, and PAINS study

All 20,800 compounds and PF-543 were subjected to drug likeness, lead likeness, and PAINS analyses. We have identified 621 hits that obeyed all the parameters of drug likeness, lead likeness, and PAINS analyses (Figure 1), but the drug PF-543 failed at two parameters, namely ghose filtration and lead likeness. The result of top 10 compounds and PF-543 fulfilling the drug likeness features is shown in Table 1.

**Table 1.** Analysis of drug like properties of top 10 compounds and PF-543 on the basis of Lipinski’s RO5.

S. no.	Name of compounds	Molecular weight (g/mol)	H-bond donor	H-bond acceptor	*A* log *P*
1	ZINC06823429	341.45	1	4	3.39
2	ZINC95421501	346.49	1	3	2.14
3	ZINC95421070	344.47	1	4	2.99
4	ZINC95421502	346.49	1	3	2.14
5	ZINC04967864	342.44	1	5	2.98
6	ZINC97084836	329.46	1	3	3.44
7	ZINC72152243	348.50	1	4	1.36
8	ZINC84976927	348.51	0	5	2.09
9	19-Norandrostenedione	272.38	0	2	3.37
10	ZINC95975458	329.46	1	3	3.50
11	Standard drug PF-543	465.20	1	4	5.89

### 3.3. ADMET screening

The selected compounds were subjected to ADMET screening which includes *in silico* prediction of human intestinal absorption (HIA), and blood brain barrier (BBB) parameters of pharmacokinetics. Out of 621 compounds, only 492 followed the HIA and BBB criteria. The compounds which show HIA levels 0, 1, 2, and 3 reflect excellent, mild, low, and very low absorption, respectively. The BBB reflects penetration level to be very large (0), large (1), normal (2), poor (3), and limitless (4). Further, it was checked for plasma protein binding (PPB) properties of ADMET. Subsequently, 230 compounds succeeded after checking on parameter of dissolvability. Only 55 molecules were successfully depicted as non-carcinogenic. Overall process of virtual screening is summarised in Figure 1.

Moreover, aforementioned compounds are tested for CYP2D6 metabolism in which 232 compounds were observed to be nonsuppressor of Cytochrome P4502D6 showing the Bayesian score of less than 0.162. After ADMET analysis, it was observed that all the 55 compounds were following all the parameters of ADMET. This information unravelled that these compounds have excellent absorption level. Additionally, 10 compounds have high penetration level and the remaining 45 compounds have medium penetration level. It was also discovered that all the 55 compounds were not bound to plasma proteins indicating good bioavailability. Each of the 55 compounds were observed to be noninhibitors of CYP2D6 suggesting that these can be utilised in the liver. In the event of solubility level, all 55 compounds were following great dissolvability levels 3 and 2. With regard to toxicity, all selected compounds were found to be non-carcinogenic and non-mutagenic. Furthermore, ADMET analysis of PF-543 was found comparable to the predicted compounds. ADMET analyses of top 10 compounds along with standard molecule PF-543 are shown in Table 2.

**Table 2.** ADMET calculation of top 10 compounds and PF-543.

S. no.	Name of compounds	HIA	BBB	PPB	CYP2D6	Solubility	Toxicity	*A* log *P*98	PSA2D
1	ZINC06823429	0	1	2	0	2	NC	3.58	52.63
2	ZINC95421501	0	2	0	0	3	NC	2.14	50.76
3	ZINC95421070	0	2	2	0	2	NC	2.99	50.76
4	ZINC95421502	0	2	0	0	3	NC	2.14	50.76
5	ZINC04967864	0	2	0	0	2	NC	3.17	63.89
6	ZINC97084836	0	1	2	0	2	NC	3.44	47.41
7	ZINC72152243	0	2	0	0	3	NC	1.36	44.82
8	ZINC84976927	0	2	0	0	3	NC	2.09	38.62
9	19-Norandrostenedione	0	1	0	0	2	NC	3.37	34.60
10	ZINC95975458	0	1	2	0	2	NC	3.50	47.41
11	Standard drug PF-543	0	1	2	0	2	NC	5.02	67.70

### 3.4. Molecular docking analysis

In order to check the binding interaction of 55 ligands with SphK1, molecular docking studies was carried out using AutoDock tools. The illustrated data uncovered that Δ*G* values of three compounds, namely ZINC06823429, ZINC95421501, and ZINC95421070 were observed to be higher than the standard drug PF-543. Moreover, ZINC06823429 was found to have greater binding energy (–11.36 kcal/mol) as compared with other drugs including PF-543 (–9.90 kcal/mol). Therefore, it is of great importance that the predicted compound namely ZINC06823429 might be used as potent inhibitor of SphK1. The docking results of top 10 compounds and standard PF-543 is summarised in Table 3.

**Table 3.** Docking analysis of top 10 compounds and comparison with known drug.

S. no.	Name of compounds	Binding energy^a^ (kcal/mol)	Ki^b^ (μM)
1	ZINC06823429	–11.36	4.74
2	ZINC95421501	–11.29	5.31
3	ZINC95421070	–11.26	5.54
4	ZINC95421502	–11.16	6.59
5	ZINC04967864	–11.02	8.30
6	ZINC97084836	–10.71	14.21
7	ZINC72152243	–10.67	15.03
8	ZINC84976927	–10.65	15.62
9	19-Norandrostenedione	–10.47	21.05
10	ZINC95975458	–10.43	22.57
11	Standard drug PF-543	–9.90	55.04

^a^ Calculated free energy of binding (Δ*G*) in kcal/mol.

^b^ Calculated inhibition constant Ki from AutoDock Tools 4.2.

#### 3.4.1. Interaction with active site residues

**Figure 2.**
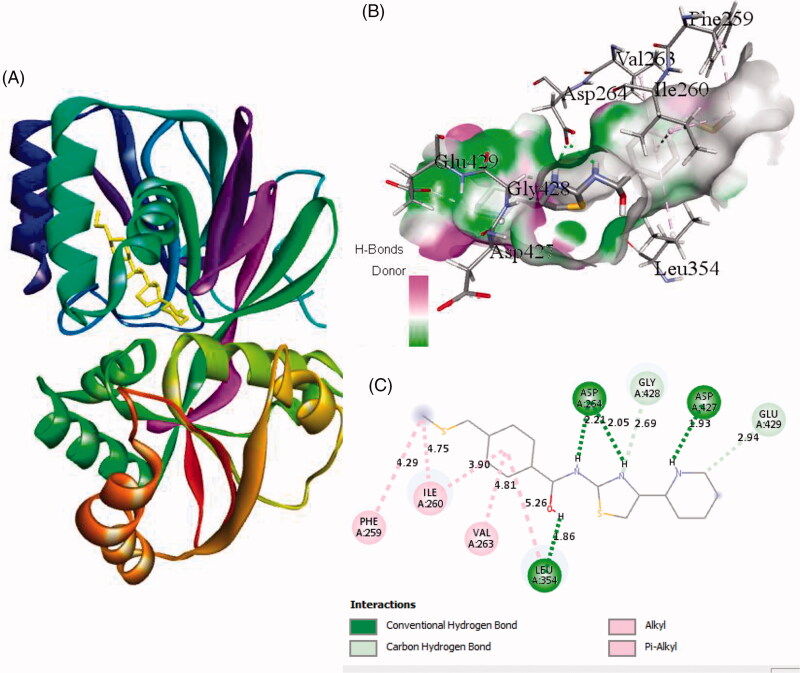
The binding mode of ZINC06823429 with the SphK1. (A) The overall structure of SphK1–ZINC06823429 complex showing protein in cartoon model and ligand in stick. (B) Interaction of ZINC06823429 to the SphK1 residues (stick). (C) 2D diagram of SphK1 interaction with the compound ZINC06823429. The active site residues of SphK1 interacting with ligand ZINC06823429 by four conventional H-bonds.

**Figure 3.**
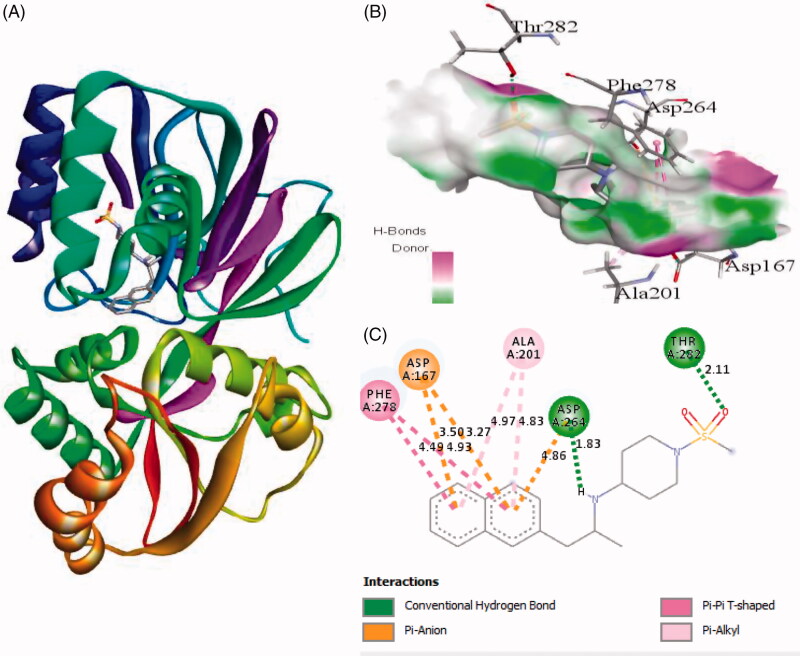
The binding mode of ZINC95421501 with the SphK1. (A) The overall structure of SphK1–ZINC95421501 complex showing protein in cartoon model and ligand in stick. (B) Interaction of ZINC95421501 to the SphK1 residues (stick). (C) 2D diagram of SphK1 interaction with the compound ZINC95421501. The active site residues of SphK1 interacting with ligand ZINC95421501 by two conventional H-bonds.

Interaction of active site residues with the three best inhibitors of SphK1 namely ZINC06823429, ZINC95421501, and ZINC95421070 was analysed and compared with standard drug PF-543. The inhibitor ZINC06823429 interacts with Phe259, Ile260, Val263, Leu354, Asp264, Gly428, Asp427, and Glu429 with distance of 4.29 Å, 4.75/3.90 Å, 4.81 Å, 5.26/1.86 Å, 2.21/2.05 Å, 2.69 Å, 1.93 Å, and 2.94 Å, respectively. It includes four conventional hydrogen bonds and several other interactions (Figure 2). In case of inhibitor ZINC95421501, the interacting residues were Phe278, Asp167, Ala201, Asp264, and Thr282 with distance of 4.49/4.93 Å, 3.50/3.27 Å, 4.97/4.84 Å, 4.86/1.83 Å, and 2.11 Å, respectively (Figure 3). It involved two conventional hydrogen bonds and several other interactions.

**Figure 4.**
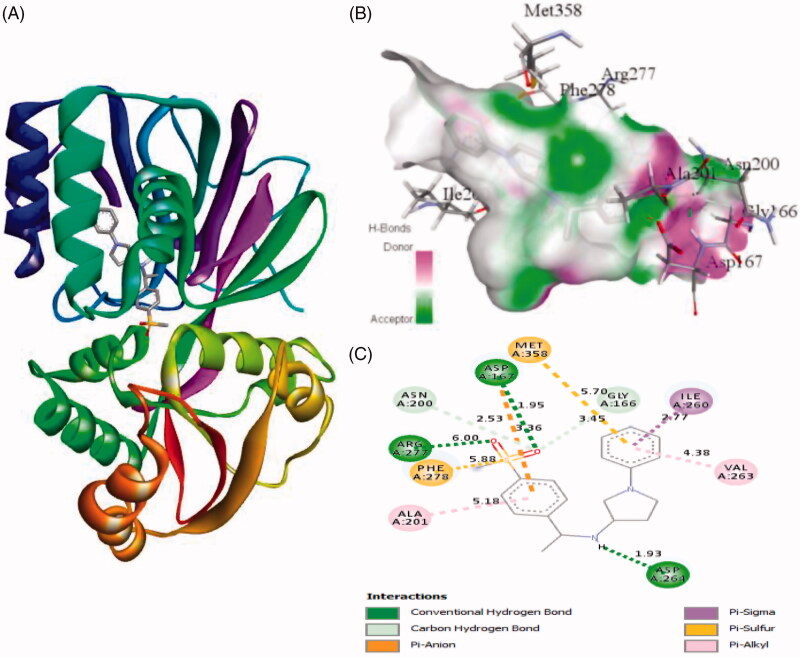
The binding mode of ZINC95421070 with the SphK1. (A) The overall structure of SphK1–ZINC95421070 complex showing protein in cartoon model and ligand in stick. (B) Interaction of ZINC95421070 to the SphK1 residues (stick). (C) 2D diagram of SphK1 interaction with the compound ZINC95421070. The active site residues of SphK1 interacting with ligand ZINC95421070 by three conventional H-bonds.

**Figure 5.**
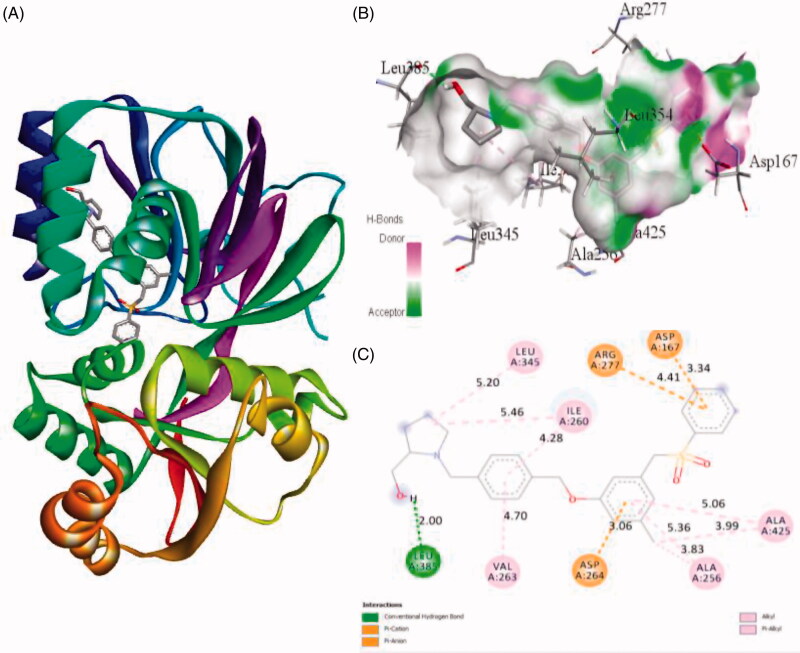
The binding mode of PF-543 with the SphK1. (A) The overall structure of SphK1–PF-543 complex showing protein in cartoon model and ligand in stick. (B) Interaction of PF-543 to the SphK1 residues (stick). (C) 2D diagram of SphK1 interaction with the compound PF-543. The active site residues of SphK1 interacting with ligand PF-543 by one conventional H-bonds.

Similarly, the inhibitor ZINC95421070 binds with the residues Ala201, Phe278, Arg277, Asn200, Asp167, Met358, Gly166, Ile260, Val263, and Asp264 with distance of 5.18 Å, 5.88 Å, 6.00 Å, 2.53 Å, 3.36/1.95 Å, 5.70 Å, 3.45 Å, 2.77 Å, 4.38 Å, and 1.93 Å, respectively (Figure 4). The inhibitor ZINC95421070 interacts with SphK1 by three conventional hydrogen bonds interactions and several other interactions. Finally, the standard drug PF-543 interacts with SphK1 with the residues Leu345, Ile260, Arg277, Asp167, Ala425, Ala256, Asp264, Val263, and Leu385 with distance of 5.20, 5.46/4.28, 4.41, 3.34, 5.06/3.99, 5.36/3.83, 3.06, 4.70, and 2.00, respectively (Figure 5). It involved only one hydrogen bond interaction. Most of the interacting residues are similar in all the docked complexes.

### 3.5. Molecular dynamics simulation

#### 3.5.1. Average potential energy of system

The average potential energy of SphK1, SphK1–PF-543, SphK1–ZINC06823429, SphK1–ZINC95421070, and SphK1–ZINC95421501 was checked prior to MD simulations to determine the stability of the systems. The constant fluctuations of temperature at 300 K for every system recommended a stable and accurate nature of MD simulations performed. An average potential energy for aforesaid system was observed to be –767,455 kJ/mol, 766,868 kJ/mol, 766,795 kJ/mol, 767,127 kJ/mol, and 766,972 kJ/mol, respectively. The potential energy for all the system was observed to be equivalent during the entire 100 ns MD process. The average potential energy of all the selected complexes is shown in Table 4.

**Table 4.** Calculated parameters for all the systems obtained after 100 ns MD simulations.

Complexes	Average potential energy (kJ/mol)	Radius of gyration (nm)	Average RMSD (nm)	Average SASA (backbone, nm^2^)	Free energy of solvation (kJ/mol/nm^2^)	Volume (nm^3^)	Density (g/l)
SphK1	–767,455	1.92	0.29	171.74	264.63	62.85	1015.11
SphK1–PF-543	–766,868	1.96	0.44	171.96	252.64	62.96	1013.30
SphK1–ZINC06823429	–766,795	1.93	0.34	168.93	255.25	63.07	1011.59
SphK1–ZINC95421070	–767,127	1.90	0.25	170.01	249.01	62.55	1019.99
SphK1–ZINC95421501	–766,972	1.92	0.27	171.82	268.47	62.94	1013.66

#### 3.5.2. Structural deviations and compactness

**Figure 6.**
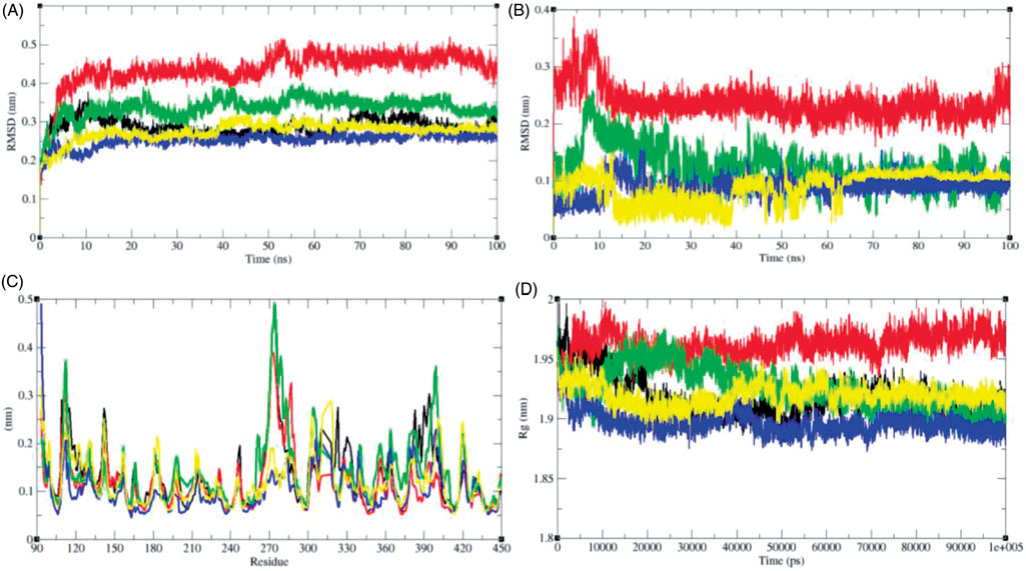
Dynamics of ligands binding to the SphK1. (A) RMSD plot as a function of time. Black, red, green, blue, and yellow colours represent values obtained for SphK1, SphK1–STD, SphK1–ZINC06823429, SphK1–ZINC95421070, and SphK1–ZINC95421501, respectively. (B) Comparison of orientations of PF-543 (red), ZINC06823429 (green), ZINC95421070 (blue), and ZINC95421501 (yellow) into the active pocket of SphK1. (C) Backbone atomic fluctuations (RMSF) plot for SphK1, SphK1–PF-543, SphK1–ZINC06823429, SphK1–ZINC95421070, and SphK1–ZINC95421501. (D) Time evolution of radius of gyration (*R*_g_) values during 100,000 ps (100 ns) of MD simulation. The *R*_g_ plot for SphK1 is shown in black. Red, green, blue, and yellow colours represent *R*_g_ plot for SphK1–PF-543, SphK1–ZINC06823429, SphK1–ZINC95421070, and SphK1–ZINC95421501, respectively.

Protein conformational changes are due to the binding of ligand in the active site of the protein^33^. Root mean square deviation (RMSD) is one of the vital parameter to determine whether the protein is stable and close to the experimental structure^63^. The average RMSD values were observed to be 0.29 nm, 0.44 nm, 0.34 nm, 0.25 nm, and 0.27 nm for SphK1, SphK1–PF-543, SphK1–ZINC06823429, SphK1–ZINC95421070, and SphK1–ZINC95421501, respectively (Table 4). The RMSD plot suggested that the binding of PF-543 to the SphK1 leads to high structural deviations from 0.29 nm to 0.44 nm. Likewise, the structural deviations from 0.29 nm to 0.34 nm were also reported in case of SphK1–ZINC06823429 (Figure 6(A)). Accordingly, the binding of ligands such as ZINC95421070 and ZINC95421501 leads to decrease in the RMSD values of SphK1. The RMSD plot suggested that inhibition of SphK1 leads to large structural deviations, and ligands PF-543 and ZINC06823429 were reported to have maximum inhibition of the SphK1. The orientations of PF-543, ZINC06823429, ZINC95421070, and ZINC95421501 were also analysed in the internal cavity of the SphK1 (Figure 6(B)). The PF-543 indicated maximum RMSD value but found to be equilibrated in the active pocket of SphK1. The ligand ZINC06823429 showed constant fluctuations throughout the MD simulations. The ligand ZINC95421070 and ZINC95421501 showed random fluctuations initially, but equilibrated after 60 ns. The RMSF of the protein upon ligand binding was plotted as function of residue number to determine the mean fluctuations of all residues during the simulation (Figure 6(C)). The graphical representation of RMSF demonstrated that the fluctuations in residues are present in SphK1 at distinct regions due to binding of these ligands. The binding of PF-543 leads to decrease in residual fluctuation at 270–280 *aa*, 300–350 *aa*, and 370–400 *aa* regions. The binding of ZINC06823429 leads to slightly increase in residual fluctuations throughout the 100 ns MD simulations. Accordingly, the binding of ZINC95421070 and ZINC95421501 decreases the residual fluctuations of SphK1. These all selected ligands showed different impact on SphK1.

Radius of gyration (*R*_g_) determines the stability of protein in a biological system and is connected to compactness of the protein. Due to less compact packing, the radius of gyration is supposed to be higher. The average *R*_g_ values for SphK1, SphK1–PF-543, SphK1–ZINC06823429, SphK1–ZINC95421070, and SphK1–ZINC95421501 were observed to be 1.92 nm, 1.96 nm, 1.93 nm, 1.90 nm, and 1.92 nm, respectively (Table 4). The average *R*_g_ values of SphK1 and SphK1–ZINC95421501 were found to be equal. The binding of PF-543 shows high values of *R*_g_ of SphK1. The binding of ZINC06823429 initially showed the high *R*_g_ values and thereafter a sudden decrease in *R*_g_ values was reported after 50 ns. The lowest *R*_g_ values were reported in case of SphK1–ZINC95421070 (Figure 6(D)).

#### 3.5.3. Solvent accessible surface area

**Figure 7.**
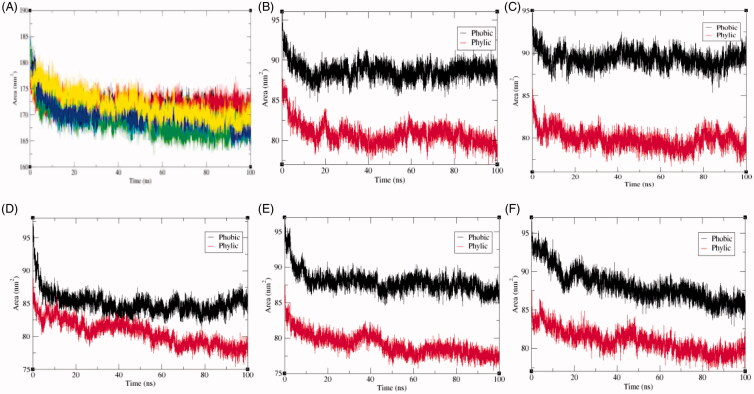
Solvent accessible surface area. (A) The solvent accessible surface area (SASA) as a function of time. Black, red, green, blue, and yellow colours represent values obtained for SphK1, SphK1–STD, SphK1–ZINC06823429, SphK1–ZINC95421070, and SphK1–ZINC95421501, respectively. The SASA plot was further resolved into hydrophobic and hydrophilic surface area for (B) SphK1, (C) SphK1–PF-543, (D) SphK1–ZINC06823429, (E) SphK1–ZINC95421070, and (F) SphK1–ZINC95421501, respectively.

**Figure 8.**
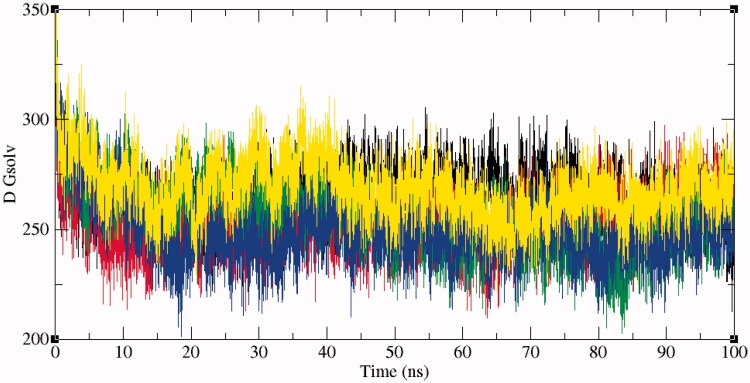
Free energy of solvation during SASA analysis. Free energy of solvation during SASA analysis was calculated for SphK1, SphK1–PF-543, SphK1–ZINC06823429, SphK1–ZINC95421070, and SphK1–ZINC95421501, respectively. Black, red, green, blue, and yellow colours represent the values obtained for SphK1, SphK1–PF-543, SphK1–ZINC06823429, SphK1–ZINC95421070, and SphK1–ZINC95421501, respectively.

The solvent accessible surface area (SASA) is characterised by the region of a protein that is accessible to solvent molecules. The average SASA values for SphK1, SphK1–PF-543, SphK1–ZINC06823429, SphK1–ZINC95421070, and SphK1–ZINC95421501 were also observed during the 100 ns MD simulations. The average SASA values for SphK1, SphK1–PF-543, SphK1–ZINC06823429, SphK1–ZINC95421070, and SphK1–ZINC95421501 were found to be 171.74 nm^2^, 171.96 nm^2^, 168.93 nm^2^, 170.01 nm^2^, and 171.82 nm^2^, respectively (Table 4). The average SASA values were comparable for all the systems except a slight decrease in SASA found in case of SphK1–ZINC06823429 and SphK1–ZINC95421070. No significant changes in SASA values were reported upon binding of PF-543 and ZINC95421501 in the active pocket of SphK1 (Figure 7). It can be inferred that the internal residues in the SphK1 are not accessible to solvent because of binding of PF-543 and ZINC95421501. The free energy of solvation of SphK1, SphK1–PF-543, SphK1–ZINC06823429, SphK1–ZINC95421070, and SphK1–ZINC95421501 was also calculated during SASA calculations. The average free energy of solvation of SphK1, SphK1–PF-543, SphK1–ZINC06823429, SphK1–ZINC95421070, and SphK1–ZINC95421501 was found to be 264.63 kJ/mol/nm^2^, 252.64 kJ/mol/nm^2^, 255.25 kJ/mol/nm^2^, 249.01 kJ/mol/nm^2^, and 268.47 kJ/mol/nm^2^, respectively (Table 4, Figure 8).

#### 3.5.4. Hydrogen bonds analysis

**Figure 9.**
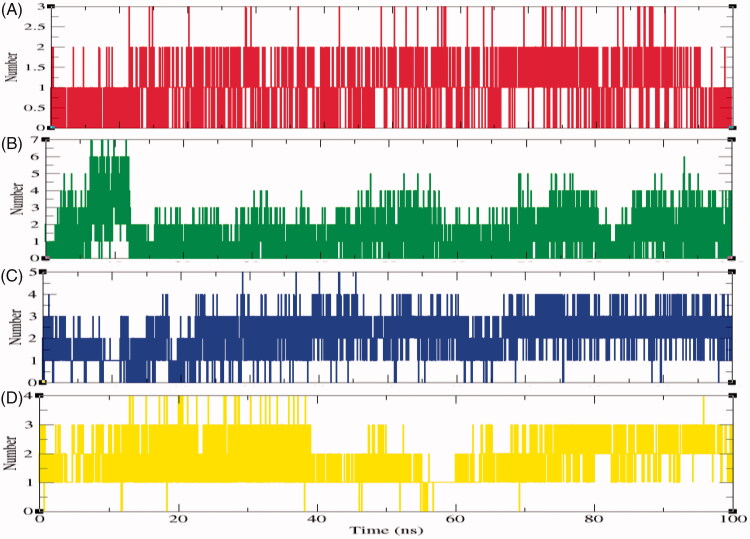
The average number of hydrogen bonds as a function of time. Red, green, blue, and yellow colours represent the number of hydrogen bonds for SphK1–PF-543, SphK1–ZINC06823429, SphK1–ZINC95421070, and SphK1–ZINC95421501, respectively.

The specificity of protein and ligand interaction is governed by hydrogen bonding which is a part of molecular recognition^64^. To validate the stability of docked complexes, the hydrogen bonds paired with 0.35 nm amongst protein and ligands in SphK1–PF-543, SphK1–ZINC06823429, SphK1–ZINC95421070, and SphK1–ZINC95421501 were computed during the 100 ns MD simulations. It has been found that ligands such as PF-543, ZINC06823429, ZINC95421070, and ZINC95421501 form maximum 2–3, 6–7, 4–5, and 3–4 hydrogen bonds with active site residues of SphK1, respectively (Figure 9). The ligand ZINC95421501 was found to have week interactions during 55–60 ns time scale.

#### 3.5.5. Secondary structure changes upon ligand binding

**Figure 10.**
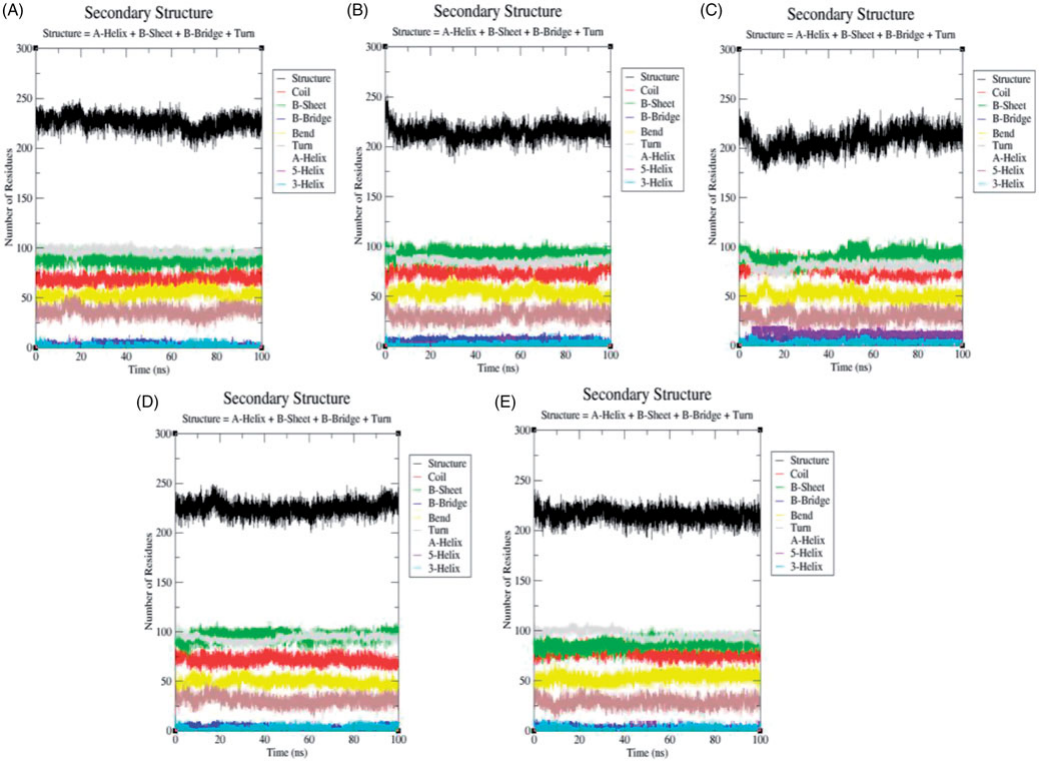
The secondary structure plot. The graphical representation indicating the structural elements present during 100 ns MD simulations in (A) SphK1, (B) SphK1–PF-543, (C) SphK1–ZINC06823429, (D) SphK1–ZINC95421070, and (E) SphK1–ZINC95421501, respectively.

**Figure 11.**
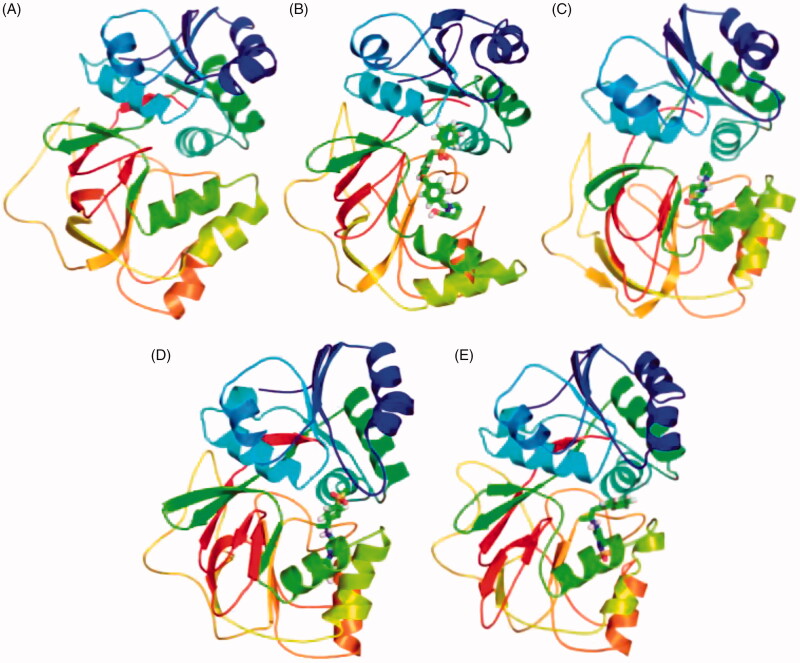
Position of ligands in the secondary structure framework. The secondary structure representation of SphK1 during 100 ns MD simulation in (A) SphK1, (B) SphK1–PF-543, (C) SphK1–ZINC06823429, (D) SphK1–ZINC95421070, and (E) SphK1–ZINC95421501, respectively.

The secondary structure components such as α-helix, β-sheet, and loops were categorised and treated as individual components for each time step. The average number of residues took part in secondary structure formation were decreased in all cases (Table 5). The binding of ligand ZINC06823429 to the SphK1 affects much than other ligands. It may be due to proper inhibition of SphK1 or unfolding in α-helical regions. The β-sheets were not affected much in case of SphK1–PF-543 and SphK1–ZINC06823429 (Figure 10). The structures of SphK1, SphK1–PF-543, SphK1–ZINC06823429, SphK1–ZINC95421070, and SphK1–ZINC95421501 were also extracted during the 100 ns MD simulations to check the position of ligands (Figure 11). The docked ligands occupied the internal pocket of SphK1 during the 100 ns time scale.

**Table 5.** Percentage of residues participated in average structure formation.

	Percentage of protein secondary structure (SS%)							
Complexes	Structure^a^	Coil	β-Sheet	β-Bridge	Bend	Turn	α-Helix	3_10_-helix
SphK1	65%	19%	26%	1%	15%	10%	28%	1%
SphK1–PF-543	61%	21%	26%	2%	15%	9%	25%	1%
SphK1–ZINC06823429	59%	22%	26%	2%	15%	8%	23%	1%
SphK1–ZINC95421070	64%	20%	28%	1%	14%	9%	27%	1%
SphK1–ZINC95421501	62%	22%	25%	1%	15%	8%	27%	1%

^a^ Structure = α-helix + β-sheet + β-bridge + turn.

#### 3.5.6. Principal component analysis

**Figure 12.**
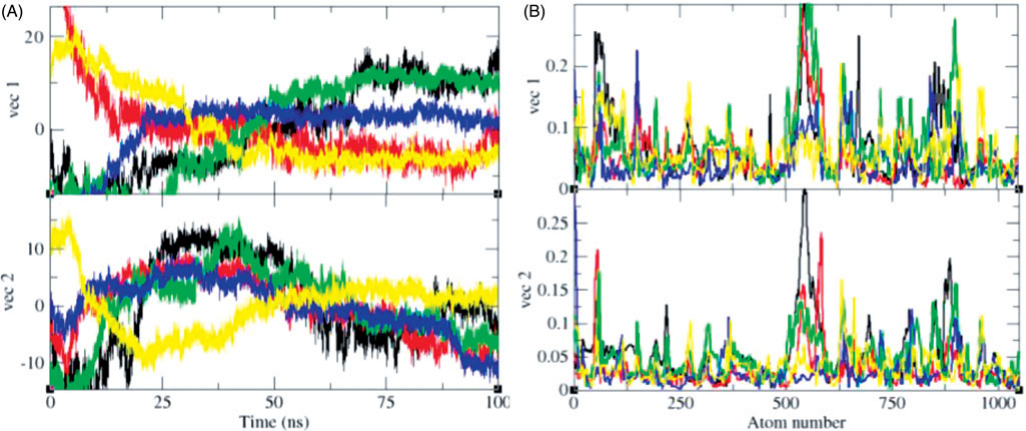
Principal component analysis (PCA). The PCA or essential dynamics (ED) calculated for SphK1, SphK1–PF-543, SphK1–ZINC06823429, SphK1–ZINC95421070, and SphK1–ZINC95421501, respectively. (A) The graph indicated the projection of eigenvectors in SphK1 (black), SphK1–PF-543 (red), SphK1–ZINC06823429 (green), SphK1–ZINC95421070 (blue), and SphK1–ZINC95421501 (yellow), respectively. (B) The Eigen root mean square fluctuations indicating the atomic fluctuations were also calculated for SphK1 (black), SphK1–PF-543 (red), SphK1–ZINC06823429 (green), SphK1–ZINC95421070 (blue), and SphK1–ZINC95421501 (yellow), respectively.

**Figure 13.**
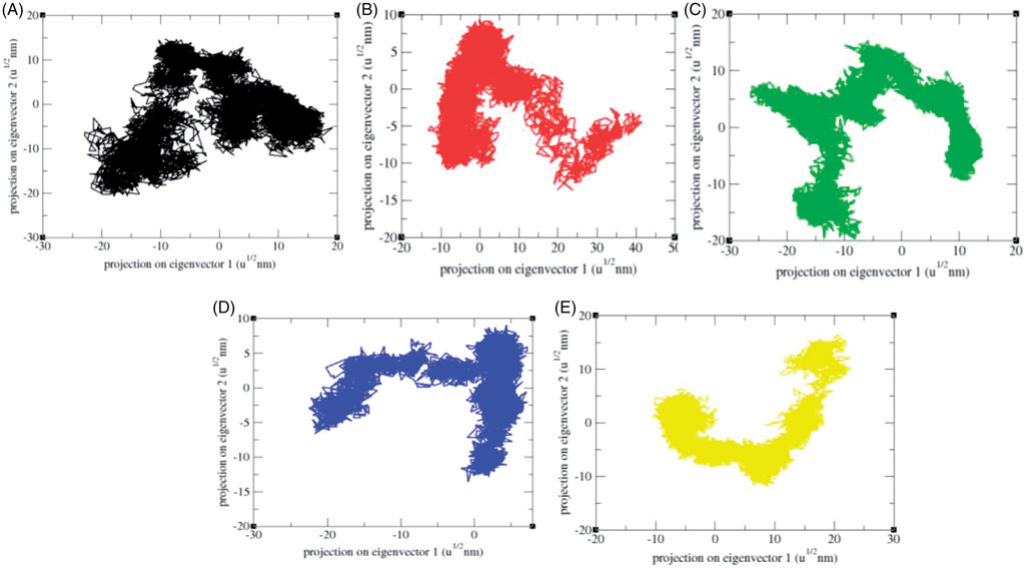
2D projection of trajectories. The 2D projections of trajectories on eigenvectors showed different projections of SphK1 in case of (A) SphK1, (B) SphK1–PF-543, (C) SphK1–ZINC06823429, (D) SphK1–ZINC95421070, and (E) SphK1–ZINC95421501, respectively.

The principal component analysis (PCA) reflects the total expansion of a protein during MD simulation^65^. The PCA for SphK1, SphK1–PF-543, SphK1–ZINC06823429, SphK1–ZINC95421070, and SphK1–ZINC95421501 was calculated. In this method, the dynamics of SphK1, SphK1–PF-543, SphK1–ZINC06823429, SphK1–ZINC95421070, and SphK1–ZINC95421501 were ascertained utilising *gmxcovar* module with reference to the backbone. PCA determines the average mobility of a protein thus revealing the internal structures responsible for atomic fluctuations^66^. The sum of eigenvalues signifies the total mobility in the system. It can be utilised to determine the flexibility of a protein under distinct conditions^67^. The trace of the covariance matrix and eigenvalues was found to be 313.94 nm^2^, 239.59 nm^2^, 341.45 nm^2^, 172.60 nm^2^, and 228.71 nm^2^ for SphK1, SphK1–PF-543, SphK1–ZINC06823429, SphK1–ZINC95421070, and SphK1–ZINC95421501, respectively. The eigenvalues were found to be increased in case of SphK1–ZINC06823429 clearly demonstrating that the irregular fluctuations in SphK1 increase due to binding of ZINC06823429. Greater eigenvalues reveal greater expansion of SphK1, i.e. low compactness and possibly denaturation. The detailed projections of eigenvectors are depicted in Figure 12. The *gmx anaeig* module reads a set of eigenvectors and eigenvalues as input and returns to project an MD trajectory along a selected eigenvector values. The atomic fluctuations during eigenvector calculation were also reported. It indicated the random fluctuations in the atoms of SphK1 upon binding of different ligands. The 2D projection of eigenvectors indicated diverse protrusions of SphK1 upon binding with different ligands molecules (Figure 13). The outcomes suggested that the distinct positions of atoms are due to conformational changes in the SphK1.

#### 3.5.7. MMPBSA analysis

The binding energies were calculated by utilising the polar and apolar solvation criterion. This investigation set up the energies related with the binding of PF-543, ZINC06823429, ZINC95421070, and ZINC95421501 to SphK1 during the 100 ns MD simulations. The following protein–ligand energies have been computed such as vdW interaction energy, electrostatic energy, polar solvation energy, SASA energy, and average binding energy (Table 6). The polar energy and apolar energy were distinct in different cases. The protein–ligand vdW energy, protein–ligand electrostatic energy, and protein–ligand total energy were found to be lowest in case of SphK1–ZINC95421070. The final average binding energy calculations also recommended the week interaction of ZINC95421070 in the internal cavity of SphK1. The ligands ZINC06823429 and PF-543 are found to have a good binding energy values.

**Table 6.** Average binding energy calculation using *g_mmpbsa* package implemented in GROMACS for high-throughput MMPBSA calculation for protein–ligand complexes.

		Polar solvation energy (kJ/mol)			Vacuum MM energy (kJ/mol)			Average binding energy (kJ/mol)		
S. no.	Complexes	Protein PB energy	LIG PB energy	Protein-LIG PB energy	Protein-LIG VdW energy	Protein-LIG Elec. energy	Protein-LIG total energy	van der Waals energy	Electrostatic energy	Binding energy
1	SphK1–PF-543	–9300	–272	–9135	–260	–430	–690	–259	–430	–276
2	SphK1–ZINC06823429	–9525	–775	–9051	–137	–1474	–1611	–138	–1474	–380
3	SphK1–ZINC95421070	–9085	–272	–9260	–40	–144	–155	–40	–115	–61
4	SphK1–ZINC95421501	–9151	–273	–8931	–219	–449	–668	–219	–449	–194

## 4. Discussion

Breast carcinoma continues to be the world’s leading cause of women’s death owing to its poor prognosis and recurrence. It has been observed that SphK1, a kinase that generates S1P, is highly elevated in multiple carcinomas including breast carcinoma^14^. The expression level of SphK1 was examined in feline mammary tumour specimens and found that SphK1 plays an important role in feline mammary tumours and may be used as therapeutic target^68^. Targeting SphK1 in triple-negative MDA-MB-231 breast cancer cells decreased proliferation and survival by compromising protein kinase C activity and cytokinesis^69^. SphK1 activity has also been linked to the effects of several microRNAs that are regulated by oestrogen receptor (ER). The miR-515-5P, a tumour suppressor shows to reduce SphK1 activity and loss of miR-515-5P. In addition, 17β-oestradiol treatment downregulated miR-515-5P levels in ER-positive compared to ER-negative breast cancers^70^. Recently, Costales et al.^71^ designed a small molecule that selectively targets the microRNA-515 hairpin precursor to inhibit the production of miR-515 that represses SphK1 enzyme.

Several preclinical studies have used mouse breast cancer models to investigate the effects of SphK1 inhibitors on tumour growth. A variety of sphingolipid based and nolipidic small molecule inhibitors of SphK1 have been reported and have shown cytotoxic effects in several resistant cancer cell lines^72^. Many of the inhibitors are withdrawn from clinical trials due to their side effects and limited efficacy. A combination of the non-specific SphK1 inhibitor SKI-II with gefitinib significantly inhibited growth of xenograft MDA-MB-468 triple-negative breast cancer (TNBC) tumours, whereas neither SKI-II nor gefitinib alone had any effects^73^. Another SphK1 inhibitor SKI-5C significantly reduced the growth of tumours from TNBC cell line MDA-MB-231 in xenografted SCID mice^74^. Recently, a series of amidine-based inhibitors with high selectivity for SphK1 were predicted using docking study^75^^,^^76^. However, these inhibitors display a short half-life. A novel sphingosine competitive inhibitor PF-543 was discovered and found to inhibit SphK1 with an inhibitory constant of 3.6 nM. In 1483 head and neck carcinoma cells, which are characterised by overexpression of SphK1 and high rate of S1P production, PF-543 decreased the level of endogenous S1P^77^. In this regard, we have chosen PF-543 as standard inhibitor of SphK1 to compare our findings. Until now, only few inhibitors are known to be effective in inhibition of SphK1 in breast carcinoma but were found to have poor pharmacokinetics profile, cytotoxic at micro molar concentration and ineffective for persistent survival rate in human trials^23^. Hence, it is required to identify more influential inhibitors that selectively inhibit SphK1 and lead to apoptosis and growth arrest in breast cancer cells without causing cytotoxic impact in healthy cells.

The computational strategies like virtual screening have been used widely as effective approaches in order to discover and develop new compounds. Previously, we have studied structural and conformational changes in SphK1 at different pH using various spectroscopic techniques and computational methods^78^. SphK1 maintains its secondary and tertiary structure in the pH range of 7.5–10.0. However, protein aggregation was observed in the acidic pH range. In the present work, we have identified a potent inhibitor of SphK1 by using several computational approaches such as LBVS, molecular docking, MD simulations, and the MMPBSA calculations. The results were compared with a known inhibitor of SphK1 called PF-543^77^. Our analysis suggested that the Log*P* value of ZINC06823429 is about 3.39 and that of PF543 is approximately 5.89, respectively. Thus, the probability of ZINC06823429 being well absorbed is much better as compared to PF-543. The crystal structures of SphK1 were reported in complex with inhibitor PF-543, that will aid in the design of better SphK1 inhibitors with improved properties^36^. Additionally, the ZINC06823429 followed all the properties of RO5 that makes it a drug like molecule. It has same number of hydrogen bond donor as well as hydrogen bond acceptor when compared with PF-543. In accordance with the ADMET profiling, the HIA, PPB, BBB, CYP2D6 solubility, and toxicity value of ZINC06823429 was found to be in harmony with the standard drug PF-543.

The molecular docking analysis suggested that ZINC06823429 has higher binding energy (–11.69 kcal/mol) than the PF-543 (–9 kcal/mol). The MD simulations study suggested that the binding of ZINC06823429 leads to unfolding of SphK1 at greater extent. This may be due to proper inhibition of SphK1 or unfolding of α-helical regions. The trace of the covariance matrix and Eigenvalues was higher in case of SphK1–ZINC06823429 than SphK1–PF-543 due to irregular fluctuations in SphK1 upon binding of ZINC06823429. Finally, the MMPBSA analysis suggested that the average binding energy of ZINC06823429 with SphK1 was highest among all the inhibitors. The average binding energy of SphK1–ZINC06823429 and SphK1–PF-543 was found to be –380 kJ/mol and –276 kJ/mol, respectively. Thus, to the best of our knowledge, this is the first computational predictions based on LBVS, molecular docking, MD simulations, and the MMPBSA calculations to identify a novel inhibitor that precisely target SphK1.

Although, we have performed detailed computational analysis of drug binding to SphK1, there are many possible research limitations. There are no experimental validations to support this computational prediction. The experimental validations of the present work are needed to support these data in order to perform proper clinical trials of identified drug. The results of computational analysis can have several variations when it is executed by different software using different force fields. Furthermore, the chemical libraries can be extended and side chain modifications of current drug are needed for better efficacy. There are remarkable improvements in the field of protein dynamics, still the validations of simulation methods and broad range of experimental observations are required^79^. Additionally, the theoretical prediction may have several variations when it comes to experimental level such as difference in binding affinity of predicted inhibitors with SphK1. The difference may arise due to large number of possible poses of an inhibitor and different folded conformations of receptor^80^.

## 5. Conclusions

The recent computational approaches were employed to study and discover new SphK1 inhibitors that could be a starting point for a promising drug candidate in the treatment of breast cancer. Various repositories of chemical compounds such as DrugBank, Ligand Expo, ChEMBL, ChEBI, GLASS, HMDB, and ZINC databases were used to perform LBVS. The screened compounds along with standard PF-543 have been taken forward to evaluate drug likeness, ADMET properties, lead likeness, and PAINS analyses. Further, docking study revealed that the ligand ZINC06823429 has greater binding energy (–11.36 kcal/mol) than PF-543 (–9.90 kcal/mol). It was subsequently strengthened by MD simulation and MMPBSA calculations to check the stability of its mode of binding and thereby inhibiting activity of SphK1. The present study predicted strong inhibitory effects of new compound that could become a starting point for future development and structure activity relationship studies for the discovery of new SphK1 inhibitors. The experimental validations are further required to support this outcome in order to perform proper clinical trials. The computational prediction may have different inhibitory effects and binding affinity of inhibitor with SphK1. The difference may arise due to large number of possible poses of an inhibitor and different folded conformations of receptor.
